# Impact of Veterinary Feed Directive Rules Changes on the Prevalence of Antibiotic Resistance Bacteria Isolated from Cecal Samples of Food-Producing Animals at US Slaughterhouses

**DOI:** 10.3390/pathogens13080631

**Published:** 2024-07-28

**Authors:** Shamim Sarkar, Chika C. Okafor

**Affiliations:** College of Veterinary Medicine, University of Tennessee, Knoxville, TN 37996, USA; shamim.sarkar08@gmail.com

**Keywords:** antimicrobial use, antimicrobial resistance, food-borne pathogens, food animal production, veterinary feed directives, United States

## Abstract

This study examined the impact of the 2017 Veterinary Feed Directive (VFD) rule changes on the prevalence of tetracycline-resistant and erythromycin-resistant bacteria (*Salmonella* spp., *Campylobacter* spp., and *Escherichia coli*) in cecal samples of food animals (cattle, swine, chicken, and turkey) at US slaughterhouses. Multivariable logistic regression was used to analyze 2013–2019 cecal samples of food-producing animals surveillance data from the National Antimicrobial Resistance Monitoring System (NARMS) in the U.S. The variables included year (used to evaluate VFD rule changes), host, and quarter of year. The analysis of surveillance data showed that the VFD rule changes have varying effects on tetracycline-resistant and erythromycin-resistant bacteria in food animals. For example, the odds of detecting tetracycline-resistant *Salmonella* spp. decreased in cattle but increased in chickens following the implementation of the VFD rule changes. Similarly, the odds of detecting tetracycline-resistant *Escherichia coli* decreased in chickens but increased in swine after the VFD rule changes. The odds of detecting erythromycin-resistant *Campylobacter* spp. increased in cattle but decreased in chickens after the VFD rule changes. In conclusion, the implementation of VFD rule changes has been beneficial in reducing the odds of detecting tetracycline-resistant *Escherichia coli* and erythromycin-resistant *Campylobacter* spp. in chickens, as well as tetracycline-resistant *Salmonella* spp. in cattle at US slaughterhouses.

## 1. Introduction

Pathogenic antimicrobial-resistant bacteria are considered public health threats [1,2]. The use of antimicrobial drugs in food-producing animals for production or growth-enhancing purposes is an important factor that increases the risk of developing antimicrobial-resistant bacteria in food-producing animals [3,4]. Medically important antimicrobial drugs are those authorized for use in human medicine [5]. These drugs are essential for treating infections and maintaining public health. Medically important antimicrobials are categorized, according to specific criteria, as either critically important (e.g., erythromycin), highly important (e.g., tetracycline), or important for human medicine [5]. The WHO has developed a medically important antimicrobials list for human medicine, which serves as a risk management tool to minimize the impact of antimicrobials use in non-human sectors such as animal farming [5].

The amount, length of time, and type of antimicrobial drugs used all play a role in developing antimicrobial-resistant bacteria [3,6,7]. Bacteria exposure to antimicrobials is associated with an increased selective proliferation of resistant bacteria [8]. Reducing inappropriate antimicrobial use in food-producing animals may improve antimicrobial-resistant prevention and control [6]. The judicious use of antimicrobials can reduce the selection pressure for developing resistant bacteria [9] in food-producing animals. An observational study demonstrated that six months after the removal of tetracycline-supplemented feed from a chicken farm, there was a lower frequency of tetracycline-resistant *E. coli* isolates compared to before the removal of the tetracycline-supplemented feed in the farm [10].

In 2017, the United States (U.S.) Food and Drug Administration (FDA) implemented the Veterinary Feed Directive (VFD) rule changes to limit the medically important antimicrobial drugs administered to food-producing animals through feed and water, allowing usage for treating illness [11]. A licensed veterinarian must supervise the use of these antimicrobial drugs under this rule; the changes to the VFD rules are an important strategy for ensuring the judicious use of medically important antimicrobials in food-producing animals in the U.S.

Violative sulfonamide and penicillin residues in the tissues of food animals have decreased at U.S. slaughter establishments following the implementation of the VFD rule changes compared to the period prior to implementation [12]. The use of antimicrobials in food-producing animals varies by antimicrobial class in the U.S. In 2020, tetracycline was the most commonly sold antimicrobial, comprising 66% (3,948,745 kg) of the total usage and 7% (433,394 kg) of macrolides in the U.S. food-producing animals [13], providing an adequate focus of analysis for the current study.

To support animal health authorities in implementing evidence-based targeted interventions, monitoring antimicrobial-resistant bacteria isolates from food-producing animals is essential to identify emerging antimicrobial-resistant bacteria. In 1997, the animal component of the National Antimicrobial Resistance Monitoring System (NARMS) was started by the Agricultural Research Service (ARS) of the U.S. Department of Agriculture (USDA) [14]. They tested *Salmonella* isolates obtained through the Pathogen Reduction/Hazard Analysis and Critical Control Point (PR/HACCP) program of the USDA Food Safety Inspection Services (FSIS) [14]. Later, in 2013, the FSIS and FDA began the cecal sampling program to monitor the antimicrobial susceptibility of bacteria in food-producing animals [14]. Samples of the cecal contents from cattle, swine, chicken, and turkey cecal samples were taken from slaughter establishments that are regulated by the FSIS. The establishments were randomly sampled in a tiered manner, depending on their slaughter volume, which resulted in a representative sample of national food animals’ production. These cecal samples were then tested for enteric bacteria, and their antimicrobial susceptibility was obtained at the FSIS laboratory [14,15].

In the U.S., foodborne illness caused by enteric bacteria pose a public health threat. Around 1 in 6 Americans are affected annually by a foodborne illness, leading to about 48 million cases, 128,000 hospitalizations, and 3000 deaths [16]. Contact with infected food-producing animals acts as a risk factor for *Salmonella* infections in humans in the U.S. [17,18]. Three types of enteric bacteria (*Salmonella* spp., *Campylobacter* spp., and *Escherichia coli*) commonly found in food-producing animals are responsible for almost 60% of these illnesses and hospitalizations in the U.S. [19], making them the primary focus of analysis for the present study.

Several studies have been conducted on the prevalence and trends of antimicrobial-resistant bacteria isolates from food-producing animals in the U.S. Most of the studies aimed to isolate and characterize antimicrobial susceptibility profiles of bacteria in food animals, using cross-sectional and retrospective study design in the U.S. [20,21,22,23,24,25]. The effect of the 2017 VFD final rule changes on the occurrence of bacteria resistant to medically important antimicrobial in cecal samples of food-producing animals at slaughter establishment is yet to be investigated in the U.S. Therefore, our study’s objective was to investigate whether the 2017 VFD final rule changes affected the occurrence of tetracycline-resistant and erythromycin-resistant bacteria (*Salmonella* spp., *Campylobacter* spp., and *Escherichia coli*) in cecal samples obtained from food-producing animals at slaughter establishments in the U.S. The findings of this study will provide evidence of the magnitude of impact VFD rule changes have had on the risk of tetracycline-resistant and erythromycin-resistant bacteria (*Salmonella* spp., *Campylobacter* spp., and *Escherichia coli*) in cecal samples obtained from food-producing animals at slaughterhouse facilities in the U.S.

## 2. Materials and Methods

### 2.1. Data Sources and Retrieval

On 21 March 2023, cecal samples of food-producing animal surveillance datasets from 2013 to 2019 were downloaded from the NARMS, which is publicly available data [26]. Under this surveillance system, cattle, swine, chicken, and turkey cecal are collected at slaughter facilities throughout the U.S. by the FDA and FSIS. The cecal samples are tested for enteric bacteria and their antimicrobial susceptibility [14]. The obtained data was transferred from Microsoft Excel (version 2019, Microsoft Corporation, Redmond, WA, USA) to STATA 17.1 (Stata Corporation, College Station, TX, USA) software for data validation.

### 2.2. Data Validation and Variables Used in the Analysis

The primary variable of interest utilized in selecting the final dataset for analysis was the presence of the minimum inhibitory concentration (MIC) values of tetracycline and erythromycin. The other selected variables for analysis in each dataset included year, month, host, and type genera of bacteria found (*Salmonella*, *Campylobacter*, and *Escherichia*). The bacteria categories analyzed in this study include *Salmonella* spp., *Escherichia coli*, and *Campylobacter* spp., which comprise their respective serotypes’ aggregation. The “year” variable was first analyzed for differences between years. Additionally, we collapsed the “year” variable into a categorical variable called “years of sampling”. This categorization was performed as follows: “2013–2014”, “2015–2016”, and “2017–2019”. We also created a dichotomous variable called “VFD rule change”, which collapsed the “year” variable into two categories: “before VFD rule change (2013–2016)” and “after VFD rule change (2017–2019)”. The “VFD rule changes” was a binary categorical variable. Cecal samples collected and tested between 2017 and 2019 were considered to be “after VFD rule changes”, whereas cecal samples collected and tested between 2013 and 2016 were considered as “before VFD rule changes” period. The “month” variable was categorized into four quarters: Quarter 1 (January, February, and March), Quarter 2 (April, May, and June), Quarter 3 (July, August, and September), and Quarter 4 (October, November, and December). The outcome variables were the presence of tetracycline-resistant *Salmonella* spp., *Campylobacter* spp., and *Escherichia coli*, as well as erythromycin-resistant *Campylobacter* spp. isolates in cecal samples from cattle, swine, chicken, and turkey.

Tetracycline-resistant *Salmonella* spp. *and Escherichia coli* isolates in cecal samples were categorized based on the MIC breakpoint values of ≥16 µg/mL. Similarly, tetracycline-resistant *Campylobacter* spp. in cecal sample was categorized based on the MIC breakpoint values of ≥4 µg/mL. Additionally, erythromycin-resistant *Campylobacter* spp. in cecal sample was defined based on the MIC breakpoint values of erythromycin (≥8 µg/mL). The breakpoints were based on the 2021 NARMS Interpretive Criteria for Susceptibility Testing [27].

### 2.3. Statistical Analysis

Frequencies and percentages were used to summarize categorical predictor variables. Four multivariable logistic regression models were built for the tetracycline-resistant *Salmonella* spp., *Campylobacter* spp., and *Escherichia coli*, as well as erythromycin-resistant *Campylobacter* isolates in cecal samples of food-producing animals (cattle, swine, chicken, and turkey) in this study. The proportion of antibiotic-resistant bacteria isolates for tetracycline and erythromycin was determined by dividing the number of resistant isolates by the total number of bacteria isolates tested for each antibiotic. The Cochran–Armitage trend tests were used to evaluate temporal trends in the proportion of cecal samples resistant to individual antibiotics, categorized by hosts, from 2013 to 2019.

For each model-building process, two steps were involved. In the first step, a univariable logistic regression model was fitted to assess the unadjusted associations between potential independent variables and the outcome variable. We conducted the univariable logistic regression analysis to investigate the association of the “year”, “years of sampling”, “VFD rule change”, host, and quarter of year with the resistant outcomes. A relaxed *p*-value ≤ 0.2 [28,29,30,31] was used to identify the predictors that were chosen for further examination in the multivariable logistic regression models in step two.

To prevent the inclusion of collinear variables in the multivariable models, the pairwise collinearity of these variables was examined using Spearman’s rank correlation coefficient. If the correlation coefficient between the two variables was ≥0.6 [32], only the variable with the higher odds ratio, the fewest missing observations in the initial screening, and biological plausibility would be included in the multivariable model.

Categorical variables with more than two levels of categories were analyzed to evaluate pairwise differences using the Tukey–Kramer adjustment for multiple comparisons.

Temporal graphs were generated in Excel (version 2019, Microsoft Corporation, Redmond, WA, USA) to visualize the temporal patterns of resistant outcomes by years of sampling.

If variables had similar characteristics, such as year, year of sampling, and VFD rule change, only one was included in the multivariable model based on the results of the univariable analysis.

In the second step, a multivariable logistic regression model was built using a manual backward elimination method. A full model was first constructed by including all the screened variables. We included the VFD rule change in every full multivariable model, regardless of the *p*-value obtained from the univariable analysis, as it was our primary exposure of interest for the analysis. Non-significant variables were removed through a manual backward elimination process. However, if the removal of a non-significant variable resulted in a substantial change (more than 20%) in the coefficient of any remaining variables in the model, it was considered a potential confounder and was retained in the final model [33]. Each final multivariable model was checked for possible multicollinearity using variance inflation factor (VIF) to avoid modeling issues associated with multicollinearity. If the VIF value exceeded 10, it indicated the presence of multicollinearity [34]. The relevant pairwise significant interaction was assessed in the final model [33]. For instance, the two-way interaction between VFD rule changes and the host categories was assessed.

All final multivariable model results were presented as an odds ratio (OR) with a corresponding 95% confidence interval (CI) and *p*-value. A *p*-value ≤ 0.05 was considered statistically significant. The overall assessment of the final multivariable model was performed using Akaike’s Information Criterion (AIC) [35]. The model with the lowest AIC values was considered the best-fitting model. Statistical analyses were performed in SAS 9.4 (SAS Institute Inc., Cary, NC, USA).

## 3. Results

The original dataset contained 54,115 records from 2013 to 2019. However, only 47,016 (before VFD rule change: 21,405; after VFD rule change: 25,521) of these records had MIC values for the target tetracycline. Additionally, only 25,430 (before the VFD rule change: 11,741; after the VFD rule change:13,689) of these records had MIC values for the target erythromycin.

### 3.1. Temporal Trends in the Proportion of Antibiotic-Resistant Bacteria

**Figure 1:**
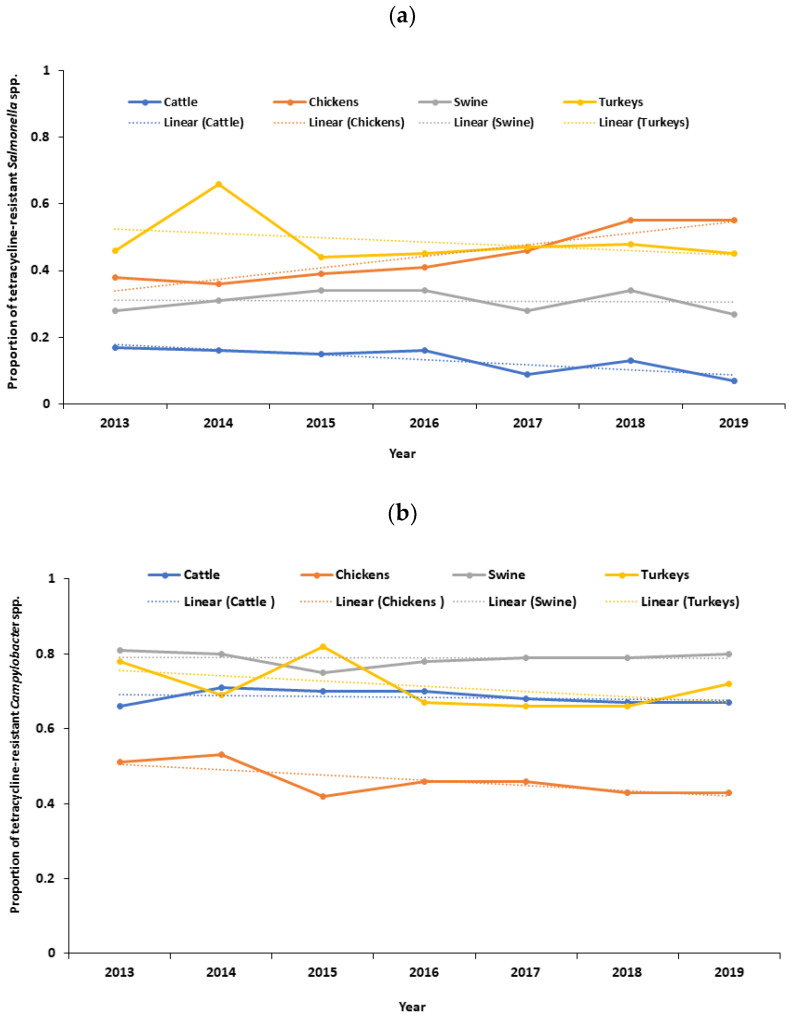

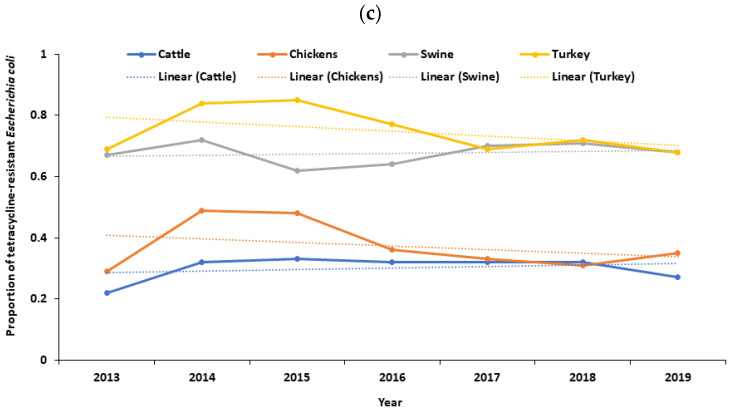
(**a**) Temporal trends in the proportion of tetracycline-resistant *Salmonella* spp. isolated from cecal samples of food animals in the United States, 2013–2019; (**b**) temporal trends in the proportion of tetracycline-resistant *Campylobacter* spp. isolated from cecal samples of food animals in the United States, 2013–2019; (**c**) temporal trends in the proportion of tetracycline-resistant *Escherichia coli* isolated from cecal samples of food animals in the United States, 2013–2019.

There was a decreasing trend in the proportion of tetracycline-resistant *Salmonella* spp. in cattle (*p* < 0.0001), with a distinct downward trend observed after 2018 (Figure 1a). Conversely, there was an increasing trend in the proportion of tetracycline-resistant *Salmonella* spp. in chickens (*p* < 0.0001) (Figure 1a). No significant trend was observed in the proportion of tetracycline-resistant *Salmonella* spp. in swine (*p* = 0.758) and turkeys (*p* = 0.259) (Figure 1a). No significant trend was observed in the proportion of tetracycline-resistant *Campylobacter* spp. in cattle (*p* = 0.334), chickens (*p* = 0.097), swine (*p* = 0.872), and turkeys (*p* = 0.930) (Figure 1b). Likewise, there was a decreasing trend in the proportion of tetracycline-resistant *Escherichia coli* in chickens (*p* = 0.014). Similarly, there was a decreasing trend in the proportion of tetracycline-resistant *Escherichia coli* in turkeys (*p* = 0.006), with a distinct downward trend after 2018 (Figure 1c). No significant trend was observed in the proportion of tetracycline-resistant *Escherichia coli* in cattle (*p* = 0.462) and swine (*p* = 0.133) (Figure 1c). Additionally, there was a decreasing trend in the proportion of erythromycin-resistant *Campylobacter* spp. in cattle (*p* = 0.002) and chickens (*p* < 0.0001), with a distinct downward trend after 2018 (Figure S1). No significant trend was observed in the proportion of erythromycin-resistant *Campylobacter* spp. in swine (*p* = 0.072) and turkeys (*p* = 0.719) (Figure S1).

### 3.2. Univariable Logistic Regression Results

Year, years of sampling, VFD rule changes, host, and the quarter of the year were significantly associated with the odds of tetracycline-resistant *Salmonella* spp. isolated in cecal samples of food-producing animals (Table 1 and Table S1). Moreover, no distinct linear pattern was observed across different categories of sampling years regarding the odds of detecting tetracycline-resistant *Salmonella* spp. in cecal samples obtained from food-producing animals (Table S1). Moreover, the graphical analysis (Figure S2) revealed no discernible linear trend in the overall proportion of tetracycline-resistant *Salmonella* spp. in the cecal samples. Similarly, year, years of sampling, VFD rule changes, and host were significantly associated with the odds of tetracycline-resistant *Campylobacter* spp. isolated from cecal samples of food-producing animals (Table 2 and Table S2). Additionally, no distinct linear pattern was observed across different categories of sampling years regarding the odds of detecting tetracycline-resistant *Campylobacter* spp. in cecal samples obtained from food-producing animals (Table S2). Moreover, the graphical analysis (Figure S3) revealed that there was no distinct linear trend in the overall proportion of tetracycline-resistant *Campylobacter* spp. in the cecal samples.

Additionally, year, host, and quarter of the year were significantly associated with the odds of tetracycline-resistant *Escherichia coli* isolated from cecal samples of food-producing animals (Table 3 and Table S3). Also, no clear linear pattern emerged across sampling years regarding the odds of detecting tetracycline-resistant *Escherichia coli* in cecal samples from food-producing animals (Table S3). Graphical analysis (Figure S4) also showed no linear trend in the overall proportion of tetracycline-resistant *Escherichia coli*. Additionally, the odds of erythromycin-resistant *Campylobacter* spp. in cecal samples were significantly associated with the year, years of sampling, host, and quarter of the year (Table 4 and Table S4). Moreover, no linear patterns were evident across sampling years of erythromycin-resistant *Campylobacter* spp. (Table S4), and graphical analysis (Figure S5) indicated no discernible trend in the overall proportion.

### 3.3. Multivariable Logistic Regression Results

The final model was fitted for tetracycline-resistant *Salmonella* spp., which included 8968 observations (Table 5). No multicollinearity issue was found in the final model. There were significant interactions between VFD rule changes and the host after controlling for all other variables in the model. The significant interaction between VFD rule changes, and the host implies that the effect of VFD rule changes on the odds of detecting tetracycline-resistant *Salmonella* spp. were not the same across the host levels. For example, the odds of detecting tetracycline-resistant *Salmonella* spp. were decreased by 41% in cattle following implementation of the VFD rule changes compared to cattle in the period prior to implementation (OR = 0.59, *p* < 0.0001) (Table 5). In contrast, the odds of detecting tetracycline-resistant *Salmonella* spp. was 1.71 times higher in chickens following the implementation of the VFD rule changes compared to chickens in the period prior to implementation (OR = 1.71, *p* < 0.0001) (Table 5). Additionally, specific to the period following implementation of the VFD rule changes, the odds of detecting tetracycline-resistant *Salmonella* spp. were decreased by 90% in cattle compared to chickens (OR = 0.10, *p* < 0.0001), 73% in cattle compared to swine (OR = 0.27, *p* < 0.0001), 87% in cattle compared to turkeys (OR = 0.13, *p* < 0.0001), and 53% in swine compared to turkeys (OR = 0.47, *p* < 0.0001) (Table 5). In contrast, the odds of detecting tetracycline-resistant *Salmonella* spp. were 2.59 times higher in chickens compared to swine (OR = 2.59, *p* < 0.0001) for the same period as above (Table 5).

The final model was fitted for tetracycline-resistant *Campylobacter*, which included 13,160 observations (Table 6). No multicollinearity issue was found in the final model. Variables significantly associated with the odds of detection of tetracycline-resistant *Campylobacter*—controlling for other variables—was the host (Table 6). There was a borderline association between the VFD rule change and the odds of detecting tetracycline-resistant *Campylobacter* isolated from cecal samples from food-producing animals (OR = 0.93, *p* = 0.0598) (Table 6).

The final model was fitted for tetracycline-resistant *Escherichia coli*, which included 12,617 observations (Table 7). No multicollinearity issue was found in the final model. Variables significantly associated with the odds of detecting tetracycline-resistant *Escherichia coli*—controlling for other variables—were VFD rule changes, host, and quarter of the year. However, there were significant interactions between VFD rule changes and the host after controlling for all other variables in the model. The significant interaction between VFD rule changes and the host implies that the effect of VFD rule changes on the odds of detecting tetracycline-resistant *Escherichia coli* were not the same across the host levels. For example, the odds of detecting tetracycline-resistant *Escherichia coli* decreased by 30% in chickens following the VFD rule changes compared to chickens prior to implementation (OR = 0.70, *p* = 0.0017) (Table 7). In contrast, the odds of detecting tetracycline-resistant *Escherichia coli* were 1.22 times higher in swine following implementation of the VFD rule changes compared to swine in the period prior to implementation (OR = 1.22, *p*= 0.0090). In addition, specific to the period following the implementation of the VFD rule changes, the odds of detecting tetracycline-resistant *Escherichia coli* were decreased by 81% in cattle compared to swine (OR = 0.19, *p* < 0.0001), 79% in chickens compared to swine (OR = 0.21, *p* < 0.0001), and 79% in chickens compared to turkeys (OR = 0.21, *p* < 0.0001) for the same period as above (Table 7).

The final model was fitted for erythromycin-resistant *Campylobacter* spp., which included 13,160 observations (Table 8). No multicollinearity issue was found in the final model. Variables significantly associated with detecting erythromycin-resistant *Campylobacter* spp.—controlling for other variables—were hosts. However, there were significant interactions between VFD rule changes and the host after controlling for all other variables in the model (Table 8). The significant interaction between VFD rule changes and the host implies that the effect of VFD rule changes on the odds of detecting tetracycline-resistant *Campylobacter* spp. were not the same across the host levels. For example, the odds of detecting erythromycin-resistant *Campylobacter* spp. were 2.68 times higher in cattle following implementation of the VFD rule changes compared to cattle in the period prior to implementation (OR = 2.68, *p* < 0.0001) (Table 8). In contrast, the odds of detecting erythromycin-resistant *Campylobacter* spp. decreased by 62% in chickens following the implementation of the VFD rule changes compared to cattle prior to implementation (OR = 0.38, *p* = 0.0005) (Table 8). Additionally, specific to the period following the implementation of the VFD rule changes, the odds of detecting erythromycin-resistant *Campylobacter* spp. was decreased by 40% in cattle compared to chickens (OR = 0.60, *p* = 0.0406), 93% in cattle compared to swine (OR = 0.07, *p* < 0.0001), 88% in chickens compared to swine (OR = 0.12, *p* < 0.0001), and 68% in chickens compared to turkeys (OR = 0.32, *p* < 0.0001) for the same period as above (Table 8).

## 4. Discussion

When studying antibiotic-resistant bacteria in food-producing animals, the factors associated with them are usually assessed independently. However, examining how the primary exposure variable interacts with other factors to the outcome variable is crucial. The present study investigated the effects of the interactions between the VFD rule changes and host categories on the odds of detecting tetracycline-resistant *Salmonella* spp., *Campylobacter* spp., and *Escherichia coli*, as well as erythromycin-resistant *Campylobacter* spp. isolated from cecal samples of food-producing animals. The present study identified the significant interactions between the VFD rule changes and host levels that imply the effect of VFD rule changes on the odds of detecting the outcome of interest were not the same across the host levels. The odds of detecting tetracycline-resistant *Salmonella* spp. were significantly decreased in cattle following the implementation of the VFD rule changes compared to cattle in the period before implementation. On the other hand, there has been a significant uptick in the odds of detecting tetracycline-resistant *Salmonella* spp. in chickens following the changes to the VFD regulations compared to the period before their implementation. Additionally, the odds of detecting tetracycline-resistant *Escherichia coli* decreased significantly in chickens following the VFD rule changes in 2017 compared to the period prior to their implementation. In contrast, the odds of detecting tetracycline-resistant *Escherichia coli* were increased significantly in swine, following the changes to the VFD regulations, compared to the prior implementation period. Moreover, the odds of detecting erythromycin-resistant *Campylobacter* spp. were significantly increased in cattle following the changes to the VFD regulations compared to the period before their implementation. In contrast, the odds of detecting erythromycin-resistant *Campylobacter* spp. were significantly decreased in chickens following the VFD rule changes compared to the period before their implementation. The results of this study can assist in directing focused research and implementing measures to mitigate the risk of the emergence of antibiotic-resistant bacteria in food-producing animals that have a higher likelihood of the emergence of antibiotic-resistant bacteria.

Implementing the VFD rule changes has significantly decreased the likelihood of detecting tetracycline-resistant *Salmonella* spp. in cattle. This can be attributed to various factors. For instance, the 2017 VFD rule changes have led to a potential decrease in tetracycline use in cattle production. A recent U.S. FDA antibiotics sales report indicates that tetracycline sales decreased in cattle production following the 2017 VFD rule changes [13]. The VFD rule changes restrict the use of medically important antibiotics, including tetracycline, for growth promotion in cattle production. It requires veterinary supervision to use tetracycline for disease prevention and control in cattle production. As a result, reduced use of tetracycline may have reduced the selective pressure of the emergence of tetracycline-resistant *Salmonella* spp. in cattle production in the U.S. [36,37,38]. A review study also reported that the reduction of antibiotic use in food-producing animals is associated with a reduction in the occurrence of antibiotic-resistant bacteria in food animals [39]. Furthermore, the observed favorable outcomes may be attributed to adopting improved biosecurity protocols, improved water, hygiene, and sanitation practices, and the utilization of vaccinations to manage infections in cattle production [40,41]. Also, after the VFD rule changes, beef and dairy operators in Tennessee (USA) reported increased interactions with licensed veterinarians [42]. Similarly, in Ohio (USA), cattle farmers reported a decrease in the use of feed antibiotics, more veterinarian–farmer interactions, and maintained record-keeping following the VFD rule changes [43]. This evidence suggests a positive link between implementing the VFD rule changes and reducing the likelihood of detecting tetracycline-resistant *Salmonella* spp. in U.S. cattle production. Additionally, we have observed a clear downward trend in the occurrence of tetracycline-resistant *Salmonella* spp. in cattle after 2018 (Figure 1a). This trend implies that the effects of the 2017 VFD rule changes have positively impacted the occurrence of tetracycline-resistant *Salmonella* spp. in cattle production within one year of implementing the rule changes. A study led by Stuart B. Levy et al. [10] observed that after six months of stopping the use of tetracycline-supplemented feed in a chicken farm, the frequency of tetracycline-resistant *Escherichia coli* decreased compared to before the feed was removed. Another study reported that avoparcin restriction regulations in Italy have decreased vancomycin-resistant enterococci found in poultry products [44]. Similarly, in the Netherlands, from 1997 to 1999, a reduction was observed in humans, broilers, and pigs following the restriction of avoparcin use [45]. The practical benefits of the VFD rule changes are evident in cattle production in the U.S. A recent review study pointed out that the European Union, notably Denmark and the Netherlands, have successfully implemented government regulations that have reduced antibiotic consumption in food animals. As a result, there has been a notable reduction in antibiotic-resistant bacteria among food animals [46].

The findings of our study indicate a higher likelihood of detecting tetracycline-resistant *Salmonella* spp. in the cecal samples of chickens. Studies have shown a relationship between antibiotic use in livestock, including chickens, and the emergence of antibiotic-resistant bacteria, which is attributed to the selective pressure exerted by antibiotics [47,48,49]. There is evidence of an association between the consumption of tetracycline and tetracycline-resistant enteric bacteria in Canadian turkey flocks [31], although the direction of association depends on the antibiotic class. It has been observed that the resistance of coliform bacteria to streptomycin in turkeys is linked to the consumption of streptomycin by the turkeys [47]. Our study results are consistent with previous findings that showed increased tetracycline-resistant *Salmonella* spp. isolates in Canadian broiler chickens after implementing the Chicken Farmers of Canada’s Antimicrobial Use Reduction Initiative [50]. Additionally, tetracycline-resistant *Salmonella* spp. in chickens can be linked to the direct and indirect exposure of tetracycline to chickens. Direct exposure to tetracycline can occur in chickens when treated with tetracycline. Tetracyclines are approved for therapeutic use in poultry production [51], including chickens in the U.S. [52]. In addition, environmental factors could also affect the occurrence of tetracycline-resistant *Salmonella* spp. in chickens by exposure to higher levels of tetracycline in the environment (via drinking water, feed, litter, feces), leading to a higher occurrence of tetracycline-resistant *Salmonella* spp. in chickens. Several studies have reported the presence of tetracycline-resistant bacteria in different farm environments. For instance, studies have reported the presence of tetracycline-resistant *Salmonella* spp. in Florida poultry litter [53] and poultry farms in the southeastern U.S. [54]. Tetracycline-resistant *Escherichia coli* has been isolated from water, sediment, and biofilms in agricultural watersheds in Canada [55]. Furthermore, tetracycline-resistant *Salmonella* spp. has been detected in poultry litter in Egypt [56]. Also, epidemiological factors could be associated with the higher odds of detecting tetracycline-resistant *Salmonella* spp. in chickens. For example, changes in the VFD rules have led to restrictions on the preventive use of tetracycline in chickens [11], which may contribute to higher odds of *Salmonella* spp. infections. The higher odds of *Salmonella* spp. infections lead to increased therapeutic use of tetracycline and selection pressure, leading to increased odds of detecting tetracycline-resistant *Salmonella* spp. in chickens. A study found that treating chickens with tetracycline led to an increase in the occurrence of tetracycline-resistant *Salmonella* spp. [57]. Another explanation can be various serovars of *Salmonella* spp. exhibit distinct resistance phenotypes, thereby implying that the distribution of serovars of *Salmonella* can have an impact on this finding [58,59,60]. Our study did not account for serovar-specific data for *Salmonella* spp. for analysis. Therefore, this limitation can be considered when interpreting overall *Salmonella* spp. Data. Another possible explanation can be the co-selection of resistance to tetracycline by exposure to other antimicrobial drugs or to chemicals (e.g., heavy metals, disinfectants) in the chicken’s farm environment may explain this finding [61]. Further research is needed to understand why tetracycline-resistant *Salmonella* spp. Increased chicken production compared to other food animal production following the VFD rule changes in the U.S.

Our study shows that the odds of detecting tetracycline-resistant *Escherichia coli* increased by 22% in the swine population after implementing the 2017 VFD rule changes. This finding can be explained by increased selection pressure due to the increasing use of tetracycline for therapeutic purposes after their restriction (as growth promoters) in swine production in the U.S. For instance, poor farm management, hygiene, and biosecurity practices can increase the chance of infectious disease occurrence. Subsequently, there is a need for the therapeutic use of antibiotics (tetracycline) in swine production in the U.S. Existing studies consistently show a clear link between increased usage of antibiotics in swine and a higher occurrence of antibiotic-resistant *Escherichia coli* [62,63,64]. A recent U.S. nationwide monitoring study has demonstrated a high frequency (34%) of tetracycline-resistant *Escherichia coli* isolates in swine at slaughter across the U.S. [25]. Future farm-level investigations could explore the factors associated with the tetracycline-resistant *Escherichia coli* as well as evaluate herd-level interventions, such as improving biosecurity measures and water, sanitation, and hygiene practices [41,65] to reduce the usage of antibiotics in U.S. swine production.

On the other hand, our study revealed a decrease in tetracycline-resistant *Escherichia coli* in chickens and turkeys. The U.S. FDA 2021 antibiotics sale report shows significant reductions in tetracycline sales in chicken and turkey production [13]. Evidence indicates a decrease in the use of tetracycline and a subsequent reduction in the prevalence of tetracycline-resistant *Escherichia coli* in broiler chickens following the implementation of the Chicken Farmers of Canada’s Antimicrobial Use Reduction Initiative [50]. There is evidence that genetic mutations of *Escherichia coli* are beneficial and prevent the induction of resistance mechanisms [66]. Further research is needed to understand this phenomenon fully.

Implementing the VFD rule changes has led to a significantly higher likelihood of detecting erythromycin-resistant *Campylobacter* spp., in the cecal samples of cattle. Several factors could explain this study’s findings. First, to treat campylobacteriosis in cattle production, erythromycin or other macrolides, such as tylosin, can be administered more frequently, as they are the preferred initial treatment [67]. The higher frequency of therapeutic use of erythromycin or other macrolide increases the selection pressure for erythromycin-resistant *Campylobacter* spp. [68] in cattle. The U.S. FDA’s recent report indicates that erythromycin sales increased in cattle production after the 2017 VFD rule changes. There is evidence of a relationship between the use of macrolides (such as tylosin and erythromycin) and the emergence of erythromycin-resistant *Campylobacters* spp. in foods of animal origin [69]. Second, increased genetic mutation in *Campylobacter* spp. could be associated with the erythromycin-resistant *Campylobacter* spp. isolated from cecal samples of cattle. There is evidence that macrolide-resistant *Campylobacter* spp. is associated with natural point mutations occurring in the peptidyl-encoding region in domain V of the 23S rRNA gene, which is the target of macrolides [70,71]. Third, *Campylobacter* spp. plasmid-mediated genetic exchanges could be associated with the erythromycin-resistant *Campylobacter* spp. isolated from cecal samples of cattle [72]. Plasmids have been reported as an important vector for the efficient spread of antibiotic-resistant phenotypes, mostly among Gram-negative bacteria, including *Campylobacter* spp. [72,73,74]. Morita et al. [75] study reported *Campylobacter* pTet plasmids are known to play an important role in the horizontal spread of antimicrobial resistance. Further farm-level studies are needed to evaluate the risk factors associated with the higher likelihood of detecting erythromycin-resistant *Campylobacter* spp. in the cecal samples of cattle.

On the contrary, the VFD rule changes were associated with lower odds of detecting erythromycin-resistant *Campylobacter* spp. in chicken cecal samples. This change in VFD rules can be attributed to the decreased utilization of erythromycin, which helps reduce the selective pressure driving the emergence of erythromycin-resistant *Campylobacter* spp. in chickens. Recent data from the U.S. FDA’s antibiotics sales report shows a decline in erythromycin sales for chicken production following the 2017 VFD rule changes [13]. Improved on-farm biosecurity, encompassing measures like sanitation, hygiene practices, and clean water access, may explain the decrease in erythromycin-resistant *Campylobacter* spp. in chickens. Such biosecurity improvements are associated with a decline in infections and a subsequent reduction in antibiotic usage. Consequently, the occurrence of erythromycin-resistant *Campylobacter* spp. in chickens experiences a positive impact. A recent systematic review highlighted that interventions, such as on-farm biosecurity and water, sanitation, and hygiene practices, can directly or indirectly lower infection frequency and minimize antibiotic usage in animal agriculture settings [41].

This study had several strengths, including a large sample size and representative sampling. It also included information about food-producing animal hosts, which helped foster an understanding of the impact of VFD rule changes on different food animal production sector such as cattle, chickens, swine, and turkeys. To test for effect modification, we analyzed the interactions of main effects with VFD rule changes and host levels. Additionally, we used multiple comparison procedures to reduce the risk of false positive statistical inference (type 1 error). Moreover, since no data were available on specific antibiotic exposure from the food animals from which cecal samples were taken, it was impossible to analyze the association between antibiotic exposure and antibiotic-resistant bacteria. Also, the absence of geographic location data may have a potential confounding effect on the outcome of these studies. Also, the absence of demographic (age, sex, and breed) and health status (apparently healthy/sick) of the sampled animals might explain differences in outcomes. Hence, we recommend that future surveillance datasets include antibiotic exposure, geographic location, demographic, and health information for enhanced statistical analysis. Additionally, to analyze the patterns of odds of detecting antibiotic-resistant bacteria in cecal samples of food-producing animals, we initially examined both the year and years of sampling using a univariable logistic regression model. Additionally, we visually assessed the linear trend of the proportion of antibiotic-resistant bacteria in cecal samples by years of sampling. However, the estimated odds ratios by years of sampling categories did not demonstrate a consistent linear pattern across all comparisons (Tables S1–S4). Furthermore, no linear trend of the overall proportion of antibiotic-resistant bacteria in cecal samples was observed based on our graphical analysis (Figures S2–S5). These non-linear relationships suggest that the impact of years of sampling on the odds of detecting antibiotic-resistant bacteria in cecal samples vary across different points of comparison. Considering these observations, we analyzed the variable “VFD rule change” as a reasonable approach. We categorized data into two groups: “before VFD rule change (2013–2016)” and “after VFD rule change (2017–2019)”. Our research question aimed to investigate significant differences in antibiotic-resistant bacteria in cecal samples, and the “VFD rule change” variable allowed us to examine the overall effect of the period after the implementation of VFD rule changes compared to the reference period (pre-VFD period: 2013–2016). Furthermore, collapsing these years into binary variables increased the category’s sample size, thereby improving the analysis’s statistical power. Furthermore, our study is constrained by a limited timeframe of only two years of post-VFD rule changes data, which limits our ability to comprehensively evaluate the long-term effects of these changes on antibiotic-resistant bacteria isolates in food-producing animals. To overcome this limitation, acquiring a dataset that encompasses a broader range of periods following the implementation of the VFD rule changes in future research endeavors is worthwhile. Despite these limitations, this study provides valuable information on whether the changes in the Veterinary Feed Directive (VFD) rule would lead to a decrease in medically important antibiotic-resistant bacteria in cecal samples obtained from food-producing animals at slaughterhouse facilities in the U.S.

## 5. Conclusions

The implementation of VFD rule changes has been beneficial in reducing the occurrence of tetracycline-resistant *Escherichia coli* and erythromycin-resistant *Campylobacter* spp. in cecal samples obtained from chickens, as well as tetracycline-resistant *Salmonella* spp. in cecal samples obtained from cattle. These findings underscore the significance of ongoing efforts to encourage the responsible and judicious use of medically important antimicrobials in food-producing animals. Such measures are crucial in combating the emergence and dissemination of antimicrobial-resistant bacteria. Nevertheless, it is important to note that there was a notable increase observed in tetracycline-resistant *Salmonella* spp. in cecal samples obtained from chickens, tetracycline-resistant *Escherichia coli* in cecal samples obtained from swine, and erythromycin-resistant *Campylobacter* spp. in cecal samples obtained from cattle. Further investigation is warranted to understand the underlying factors contributing to the rise of specific antimicrobial-resistant bacteria in particular groups of food-producing animals following the implementation of VFD rule changes in the U.S.

## Figures and Tables

**Table 1:** Results of univariable logistic regression analysis for tetracycline-resistant *Salmonella* spp. isolated from cecal samples of food-producing animals in the United States, 2013–2019.

Variable	Category	Tetracycline		OR	95% CI	*p*-Values
		Resistant n (%)	No Resistant n (%)			
Year (n = 8968)						<0.001
	2013	261 (24.42)	808 (75.58)	0.87	0.73, 1.05	0.162
	2014	306 (28.71)	760 (71.29)	1.09	0.91, 1.30	0.311
	2015	283 (28.08)	725 (71.92)	1.06	0.88, 1.27	0.513
	2016	339 (29.22)	821 (70.78)	1.12	0.94, 1.33	0.184
	2017	393 (26.88)	1069 (73.12)	Referent		
	2018	549 (33.74)	1078 (66.26)	1.38	1.18, 1.61	<0.001
	2019	453 (28.74)	1123 (71.26)	1.09	0.93, 1.28	0.253
VFD rule changes (n = 8968)						0.0176
	Before VFD rule changes (2013–2016)	1189 (27.63)	3114 (72.37)	Referent		
	After VFD rule changes (2017–2019)	1395 (29.90)	3270 (70.10)	1.11	1.01, 1.22	0.018
Host (n = 8968)						<0.001
	Cattle	376 (13.11)	2491 (86.89)	0.15	0.12, 0.20	<0.001
	Chickens	749 (48.64)	791 (51.36)	0.98	0.77, 1.25	0.891
	Swine	1303 (30.71)	2940 (69.29)	0.46	0.36, 0.57	<0.001
	Turkeys	156 (49.06)	162 (50.94)	Referent		
Quarter of year (n = 8965)						0.0008
	Quarter 1	653 (31.61)	1413 (68.39)	1.25	1.10, 1.42	<0.001
	Quarter 2	665 (29.04)	1625 (70.96)	1.11	0.97, 1.26	0.101
	Quarter 3	679 (26.91)	1844 (73.09)	Referent		
	Quarter 4	586 (28.09)	1500 (71.91)	1.06	0.93, 1.20	0.372

OR: odds ratio; CI: confidence intervals.

**Table 2:** Results of univariable logistic regression analysis for tetracycline-resistant *Campylobacter* spp. isolated from cecal samples of food-producing animals in the United States, 2013–2019.

Variable	Category	Tetracycline		OR	95% CI	*p*-Value
		Resistant n (%)	No Resistant n (%)			
Year (n = 13,160)						<0.001
	2013	1195 (68.52)	549 (31.48)	1.05	0.92, 1.21	0.440
	2014	1184 (72.37)	452 (27.63)	1.27	1.10, 1.46	0.001
	2015	1021 (70.17)	434 (29.83)	1.14	0.98, 1.31	0.077
	2016	1023 (70.45)	429 (29.55)	1.15	0.99, 1.33	0.051
	2017	1361 (67.34)	660 (32.66)	Referent		
	2018	1465 (64.17)	818 (35.83)	0.86	0.76, 0.98	0.029
	2019	1657 (64.50)	912 (35.50)	0.88	0.77, 0.99	0.044
VFD rule changes (n = 13,160)						<0.001
	Before VFD rule changes (2013–2016)	4423 (70.35)	1864 (29.65)	Referent		
	After VFD rule changes (2017–2019)	4483 (65.23)	2390 (34.77)	0.79	0.73, 0.85	<0.001
Host (n = 13,160)						<0.001
	Cattle	6131 (68.38)	2835 (31.62)	0.93	0.77, 1.13	0.515
	Chickens	630 (44.30)	792 (55.70)	0.34	0.27, 0.42	<0.001
	Swine	1792 (79.08)	474 (20.92)	1.63	1.32, 2.03	<0.001
	Turkeys	353 (69.76)	153 (30.24)	Referent		
Quarter of year (n = 13,157)						0.162
	Quarter 1	2411 (68.07)	1131 (31.93)	1.06	0.95, 1.17	0.290
	Quarter 2	2393 (67.93)	1130 (32.07)	1.05	0.94, 1.16	0.351
	Quarter 3	1983 (66.84)	984 (33.16)	Referent		
	Quarter 4	2117 (67.74)	1008 (32.26)	1.04	0.93, 1.15	0.450

OR: odds ratio; CI: confidence intervals.

**Table 3:** Results of univariable logistic regression analysis for tetracycline-resistant *Escherichia coli* isolated in cecal samples of food-producing animals in the United States, 2013–2019.

Variable	Category	Tetracycline		OR	95% CI	*p*-Value
		Resistant n (%)	No Resistant n (%)			
Year (n = 12,618)						<0.001
	2013	313 (36.23)	551 (63.77)	0.73	0.62, 0.86	<0.001
	2014	432 (47.95)	469 (52.05)	1.19	1.02, 1.39	0.025
	2015	712 (45.41)	856 (54.59)	1.07	0.94, 1.22	0.256
	2016	935 (44.15)	1183 (55.85)	1.02	0.90, 1.15	0.700
	2017	1034 (43.57)	1339 (56.43)	Referent		
	2018	1105 (43.42)	1440 (56.58)	0.99	0.88, 1.11	0.913
	2019	952 (42.33)	1297 (57.67)	0.95	0.84, 1.06	0.393
VFD rule changes (n = 12,618)						0.397
	Before VFD rule changes (2013–2016)	2392 (43.88)	3059 (56.12)	Referent		
	After VFD rule changes (2017–2019)	3091 (43.13)	4076 (56.87)	0.96	0.90, 1.04	0.398
Host (n = 12,618)						<0.001
	Cattle	2188 (30.65)	4950 (69.35)	0.16	0.13, 0.18	<0.001
	Chickens	513 (35.77)	921 (64.23)	0.20	0.16, 0.24	<0.001
	Swine	2189 (67.62)	1048 (32.38)	0.76	0.64, 0.90	0.002
	Turkeys	593 (73.30)	216 (26.70)	Referent		
Quarter of year (n = 12,617)						0.0005
	Quarter 1	1433 (46.33)	1660 (53.67)	1.20	1.09, 1.33	<0.001
	Quarter 2	1410 (42.22)	1930 (57.78)	1.02	0.92, 1.12	0.668
	Quarter 3	1358 (41.69)	1899 (58.31)			
	Quarter 4	1282 (43.80)	1645 (56.20)	1.08	0.98, 1.20	0.095

OR: odds ratio; CI: confidence intervals.

**Table 4:** Results of univariable logistic regression analysis for erythromycin-resistant *Campylobacter* spp. isolated in cecal samples of food-producing animals in the United States, 2013–2019.

Variable	Category	Erythromycin		OR	95% CI	*p*-Value
		Resistant n (%)	No Resistant n (%)			
Year (n = 13,160)						0.0011
	2013	109 (6.25)	1635 (93.75)	0.92	0.71, 1.19	0.552
	2014	125 (7.64)	1511 (92.36)	1.14	0.89, 1.47	0.287
	2015	69 (4.74)	1386 (95.26)	0.69	0.51, 0.92	0.015
	2016	72 (4.96)	1380 (95.04)	0.72	0.53, 0.97	0.031
	2017	136 (6.73)	1885 (93.27)	Referent		
	2018	144 (6.31)	2139 (93.69)	0.93	0.73, 1.18	0.576
	2019	127 (4.94)	2442 (95.06)	0.72	0.56, 0.92	0.010
VFD rule changes (n = 13,160)						0.917
	Before VFD rule changes (2013–2016)	375 (5.96)	5912 (94.04)	Referent		
	After VFD rule changes (2017–2019)	407 (5.92)	6466 (94.08)	0.99	0.85, 1.14	0.917
Host (n = 13,160)						<0.001
	Cattle	128 (1.43)	8838 (98.57)	0.12	0.08, 0.17	<0.001
	Chickens	62 (4.36)	1360 (95.64)	0.38	0.26, 0.57	<0.001
	Swine	539(23.79)	1727 (76.21)	2.66	1.97, 3.60	<0.001
	Turkeys	53 (10.47)	453 (89.53)	Referent		
Quarter of year (n = 13,160)						0.0007
	Quarter 1	224 (6.32)	3319 (93.68)	1.36	1.09, 1.69	0.005
	Quarter 2	197 (5.59)	3327 (94.41)	1.19	0.95, 1.49	0.115
	Quarter 3	140 (4.72)	2828 (95.28)	Referent		
	Quarter 4	221 (7.07)	2904 (92.93)	1.53	1.23, 1.91	<0.001

OR: odds ratio; CI: confidence intervals.

**Table 5:** Final multivariable model of factors associated with tetracycline-resistant *Salmonella* spp. isolated in cecal samples of food-producing animals (n= 8968) in the United States, 2013–2019.

Variable	Category	OR	95% CI	*p*-Value
VFD rule changes				0.3736
	After VFD rule changes (2017–2019) vs. Before VFD rule changes (2013–2016)	0.94	0.82, 1.08	0.3736
Host				<0.0001
	Cattle vs. Turkey	0.15	0.11, 0.21	<0.0001
	Chickens vs. Turkey	0.86	0.62, 1.20	0.6518
	Swine vs. Turkey	0.46	0.34, 0.62	<0.0001
VFD rule changes × Host				<0.0001
Cattle				
	After VFD rule changes vs. before VFD rule changes	0.59	0.47, 0.74	<0.0001
Chickens				
	After VFD rule changes vs. before VFD rule changes	1.71	1.36, 2.15	<0.0001
Swine				
	After VFD rule changes vs. before VFD rule changes	0.91	0.8, 1.04	0.1699
Turkey				
	After VFD rule changes vs. before VFD rule changes	0.84	0.54, 1.31	0.4473
Before VFD rule changes				
	Cattle vs. Turkeys	0.18	0.11, 0.29	<0.0001
	Chickens vs. Turkeys	0.61	0.37, 0.99	0.0479
	Swine vs. Turkeys	0.44	0.28, 0.68	<0.0001
	Cattle vs. Chickens	0.30	0.22, 0.41	<0.0001
	Cattle vs. Swine	0.41	0.33, 0.51	<0.0001
	Chicken vs. Swine	1.39	1.05, 1.84	0.0159
After VFD rule changes				
	Cattle vs. Turkeys	0.13	0.08, 0.20	<0.0001
	Chicken vs. Turkeys	1.23	0.81, 1.87	0.5922
	Swine vs. Turkeys	0.47	0.31, 0.72	<0.0001
	Cattle vs. Chickens	0.10	0.08, 0.14	<0.0001
	Cattle vs. Swine	0.27	0.21, 0.35	<0.0001
	Chicken vs. Swine	2.59	2.12, 3.16	<0.0001

OR: odds ratio; CI: confidence intervals.

**Table 6:** Final multivariable model of factors associated with tetracycline-resistant *Campylobacter* spp. isolated in cecal samples of food animals (n = 13,160) in the United States, 2013–2019.

Variable	Categories	OR	95% CI	*p*-Value
VFD rule changes				0.2258
	After the VFD rule changes (2017–2019) vs. Before VFD rule changes (2013–2016)	0.897	0.75, 1.00	1.069
Host				<0.0001
	Cattle vs. Turkeys	0.87	0.58, 1.29	0.7969
	Chickens vs. Turkeys	0.34	0.22, 0.53	<0.0001
	Swine vs. Turkeys	1.52	1.00, 2.31	0.0502
	Cattle vs. chicken	2.55	2.11, 3.09	<0.0001
	Cattle vs. Swine	0.57	0.49, 0.66	<0.0001
	Chicken vs. Swine	0.223	0.18, 0.28	<0.0001
VFD rule changes × Host				0.6049
Cattle				
	After VFD rule changes vs. before VFD rule changes	0.92	0.84, 1.01	0.0767
Chicken				
	After VFD rule changes vs. before VFD rule changes	0.83	0.63, 1.09	0.1856
Swine				
	After VFD rule changes vs. before VFD rule changes	1.03	0.84, 1.26	0.7633
Turkey				
	After VFD rule changes vs. before VFD rule changes	0.82	0.44, 1.50	0.5218
Before VFD rule changes				
	Cattle vs. Turkeys	0.82	0.38, 1.74	0.9018
	Chickens vs. Turkeys	0.34	0.15, 0.77	0.0037
	Swine vs. Turkeys	1.36	0.63, 2.93	0.7410
	Cattle vs. Chickens	2.42	1.72, 3.39	<0.0001
	Cattle vs. Swine	0.60	0.49, 0.73	<0.0001
	Chicken vs. Swine	0.25	0.17, 0.36	<0.0001
After VFD rule changes				
	Cattle vs. Turkeys	0.92	0.69, 1.21	0.8628
	Chickens vs. Turkeys	0.34	0.25, 0.46	<0.0001
	Swine vs. Turkeys	1.71	1.22, 2.37	0.0002
	Cattle vs. Chickens	2.69	2.26, 3.2	<0.0001
	Cattle vs. Swine	0.54	0.43, 0.67	<0.0001
	Chicken vs. Swine	0.20	0.16, 0.26	<0.0001

OR: odds ratio; CI: confidence intervals.

**Table 7:** Final multivariable model of factors associated with tetracycline-resistant *Escherichia coli* isolated in cecal samples of food-producing animals (n = 12,617) in the United States, 2013–2019.

Variable	Categories	OR	95% CI	*p*-Value
VFD rule changes				0.0019
	After the VFD rule changes (2017–2019) vs. Before VFD rule changes (2013–2016)	0.834	0.74, 0.94	0.0019
Host				<0.0001
	Cattle vs. Turkeys	0.14	0.11, 0.18	<0.0001
	Chickens vs. Turkeys	0.19	0.14, 0.25	<0.0001
	Swine vs. Turkeys	0.68	0.53, 0.87	0.0004
Quarter of year				0.0015
	Quarter 1 vs. Quarter 3	1.23	1.07, 1.42	0.0008
	Quarter 2 vs. Quarter 3	1.06	0.92, 1.22	0.7011
	Quarter 4 vs. Quarter 3	1.08	0.94, 1.25	0.4762
	Quarter 1 vs. Quarter 2	1.16	1.01, 1.33	0.0279
	Quarter 1 vs. Quarter 4	1.14	0.98, 1.31	0.1012
	Quarter 2 vs. Quarter 4	0.98	0.85, 1.13	0.9794
VFD rule changes × Host				<0.0001
Cattle				
	After VFD rule changes vs. before VFD rule changes	0.99	0.89, 1.09	0.7760
Chickens				
	After VFD rule changes vs. before VFD rule changes	0.70	0.56, 0.87	0.0017
Swine				
	After VFD rule changes vs. before VFD rule changes	1.22	1.05, 1.41	0.0090
Turkeys				
	After VFD rule changes vs. before VFD rule changes	0.58	0.41, 0.82	0.0025
Before VFD rule changes				
	Cattle vs. Turkeys	0.11	0.07, 0.17	<0.0001
	Chickens vs. Turkeys	0.17	0.11, 0.28	<0.0001
	Swine vs. Turkeys	0.47	0.31, 0.71	<0.0001
	Cattle vs. Chickens	0.64	0.49, 0.82	<0.0001
	Cattle vs. Swine	0.24	0.20, 0.28	<0.0001
	Chickens vs. Swine	0.37	0.28, 0.49	<0.0001
After VFD rule changes				
	Cattle vs. Turkeys	0.19	0.15, 0.24	<0.0001
	Chickens vs. Turkeys	0.21	0.16, 0.28	<0.0001
	Swine vs. Turkeys	0.98	0.75, 1.30	0.9989
	Cattle vs. Chickens	0.90	0.74, 1.10	0.5258
	Cattle vs. Swine	0.19	0.16, 0.22	<0.0001
	Chickens vs. Swine	0.21	0.17, 0.27	<0.0001

OR: odds ratio; CI: confidence intervals.

**Table 8:** Final multivariable model of factors associated with erythromycin-resistant *Campylobacter* spp. isolated in cecal samples of food animals (n = 13,160) in the United States, 2013–2019.

Variable	Categories	OR	95% CI	*p*-Value
VFD rule changes				0.4690
	After the VFD rule changes (2017–2019) vs. Before VFD rule changes (2013–2016)	0.91	0.70, 1.18	0.4690
Host				<0.0001
	Cattle vs. Turkeys	0.10	0.06, 0.18	<0.0001
	Chickens vs. Turkeys	0.44	0.24, 0.84	0.0060
	Swine vs. Turkeys	2.36	1.37, 4.07	0.0003
VFD rule changes × Host				<0.0001
Cattle				
	After VFD rule changes vs. before VFD rule changes	2.68	1.83, 3.93	<0.0001
Chickens				
	After VFD rule changes vs. before VFD rule changes	0.38	0.22, 0.66	0.0005
Swine				
	After VFD rule changes vs. before VFD rule changes	0.91	0.75, 1.10	0.3232
Turkeys				
	After VFD rule changes vs. before VFD rule changes	0.73	0.33, 1.63	0.4428
Before VFD rule changes				
	Cattle vs. Turkeys	0.05	0.02, 0.15	<0.0001
	Chickens vs. Turkeys	0.62	0.20, 1.92	0.6910
	Swine vs. Turkeys	2.12	0.79, 5.70	0.2082
	Cattle vs. Chickens	0.09	0.04, 0.18	<0.0001
	Cattle vs. Swine	0.03	0.02, 0.04	<0.0001
	Chickens vs. Swine	0.29	0.16, 0.53	<0.0001
After VFD rule changes				
	Cattle vs. Turkeys	0.19	0.12, 0.31	<0.0001
	Chickens vs. Turkeys	0.32	0.18, 0.57	<0.0001
	Swine vs. Turkeys	2.63	1.68, 4.12	<0.0001
	Cattle vs. Chickens	0.60	0.37, 0.99	0.0406
	Cattle vs. Swine	0.07	0.05, 0.10	<0.0001
	Chickens vs. Swine	0.12	0.08, 0.19	<0.0001

OR: odds ratio; CI: confidence intervals.

## Data Availability

Data used in this study are publicly available and can be accessed at: https://www.fda.gov/animal-veterinary/national-antimicrobial-resistance-monitoring-system/integrated-reportssummaries (accessed on 21 March 2023).

## Supplementary Materials

The following supporting information can be downloaded at: https://www.mdpi.com/article/10.3390/pathogens13080631/s1, Figure S1: Temporal trends in the proportion of erythromycin-resistant *Campylobacter* spp. isolated from cecal samples of food animals in the United States, 2013–2019. Figure S2: Temporal trends in the proportion of tetracycline-resistant *Salmonella* spp. by years of sampling. Figure S3: Temporal trends in the proportion of tetracycline-resistant *Campylobacter* spp. by years of sampling. Figure S4: Temporal trends in the proportion of tetracycline-resistant *Escherichia coli* by years of sampling. Figure S5: Temporal trends in the proportion of erythromycin-resistant *Campylobacter* spp. by years of sampling. Table S1: Univariable logistic regression of association between years of sampling and tetracycline-resistant *Salmonella* spp. isolates in cecal samples of food-producing animals in the United States. Table S2: Univariable logistic regression of association between years of sampling and tetracycline-resistant *Campylobacter* spp. isolates in cecal samples of food-producing animals in the United States. Table S3: Univariable logistic regression of association between years of sampling and tetracycline-resistant *Escherichia coli* isolates in cecal samples of food-producing animals in the United States. Table S4: Univariable logistic regression of association between years of sampling and erythromycin-resistant *Campylobacter* spp. isolates in cecal samples of food-producing animals in the United States.
