# Supplementary material for: Impact of Veterinary Feed Directive Rules Changes on the Prevalence of Antibiotic Resistance Bacteria Isolated from Cecal Samples of Food-Producing Animals at US Slaughterhouses
Source: Pathogens. 2024 Jul 28;13(8):631. doi: 10.3390/pathogens13080631 (PMC11357339; doi:10.3390/pathogens13080631)
Supplement: Supplementary file 1 [file pathogens-13-00631-s001.zip › pathogens-3105789-supplementary.pdf]

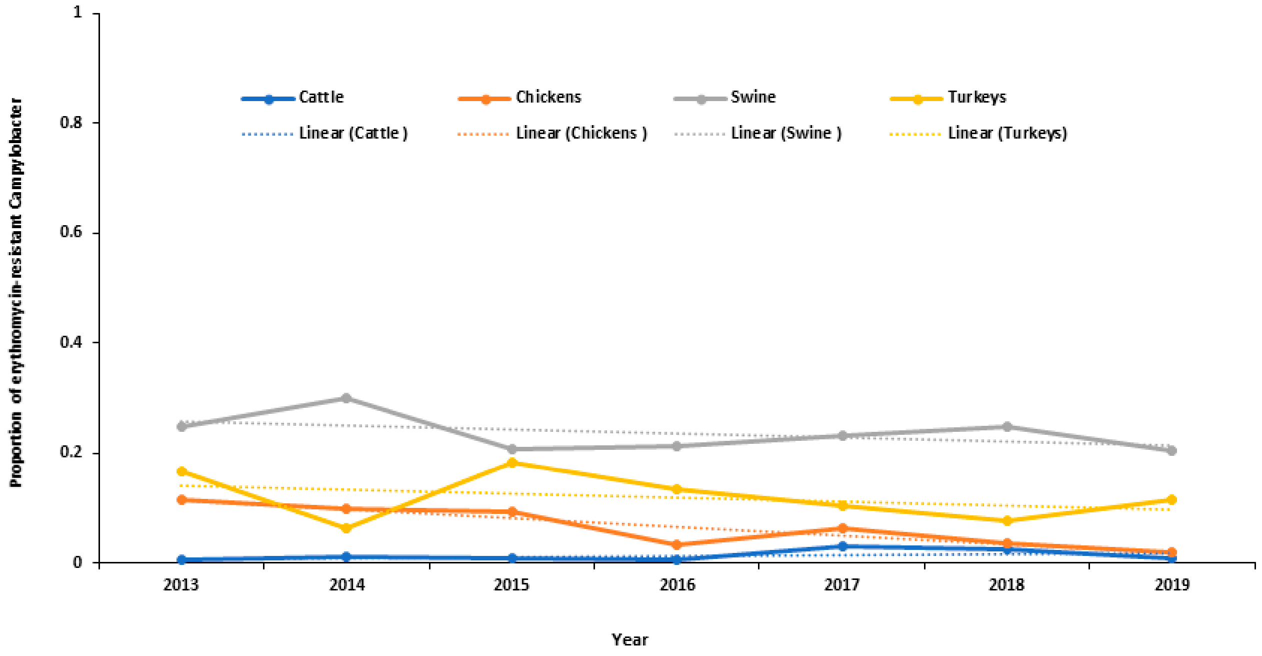

Fig. S1. Temporal trends in the proportion of erythromycin-resistant *Campylobacter* spp. isolated from cecal samples of food animals in the United States, 2013-2019

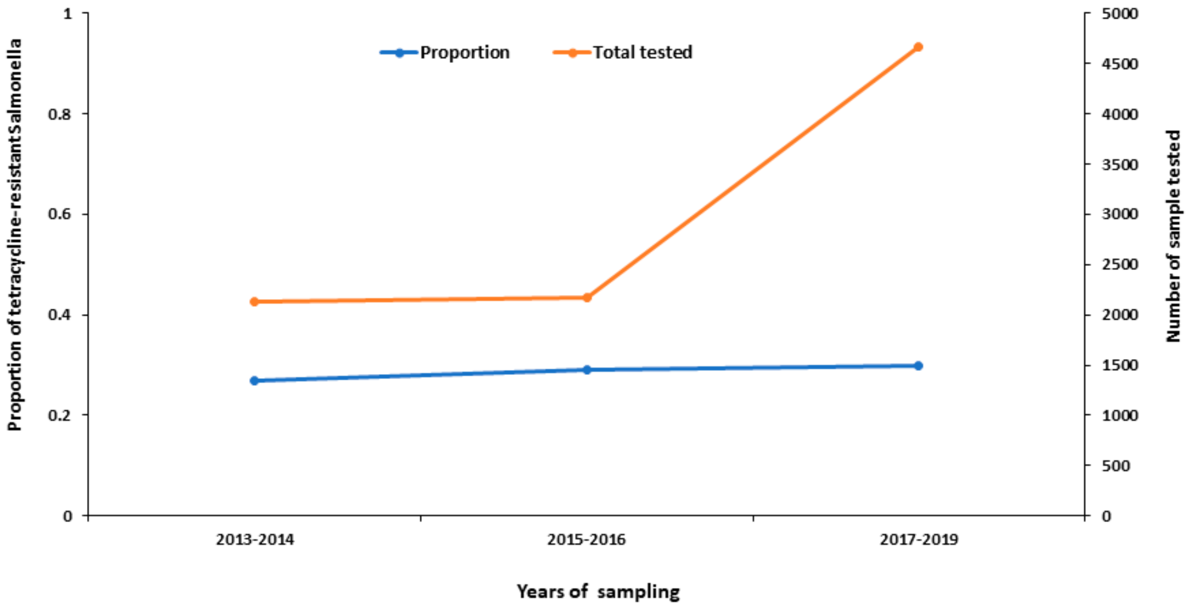

Fig. S2. Temporal trends in the proportion of tetracycline-resistant *Salmonella* spp. by years of sampling

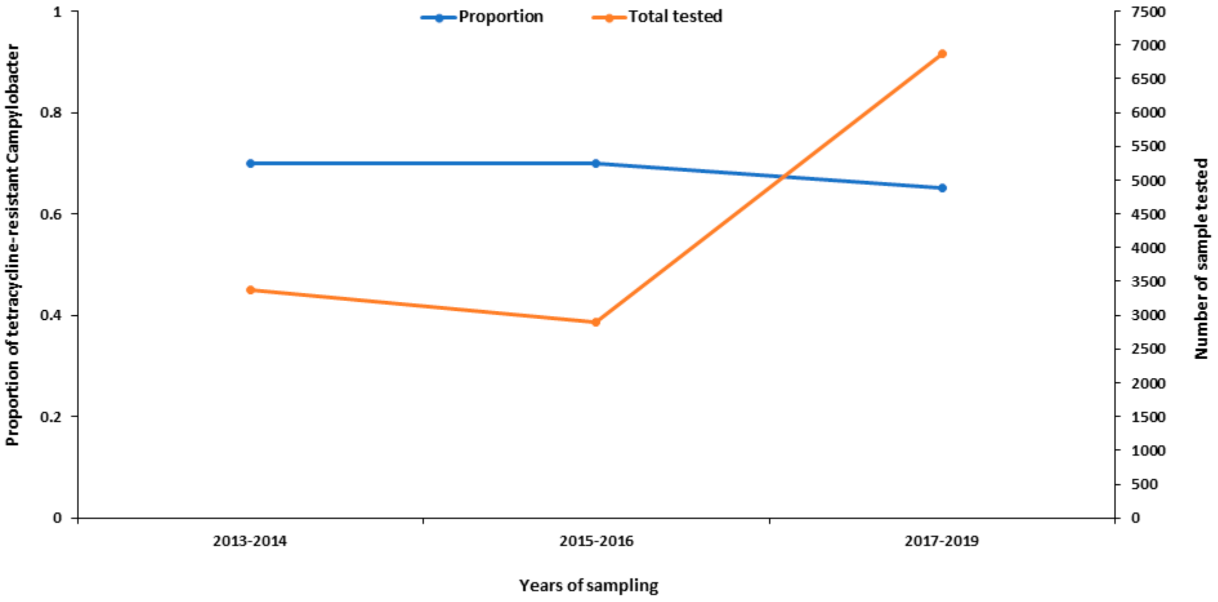

Fig. S3. Temporal trends in the proportion of tetracycline-resistant *Campylobacter* spp. by years of sampling

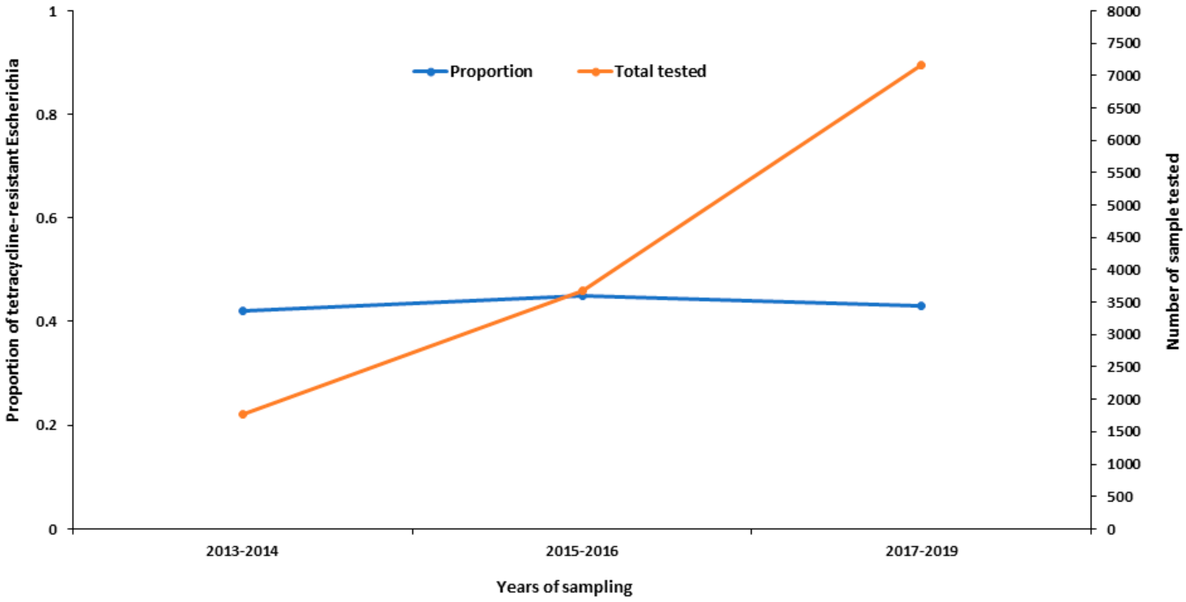

Fig. S4. Temporal trends in the proportion of tetracycline-resistant *Escherichia coli* by years of sampling

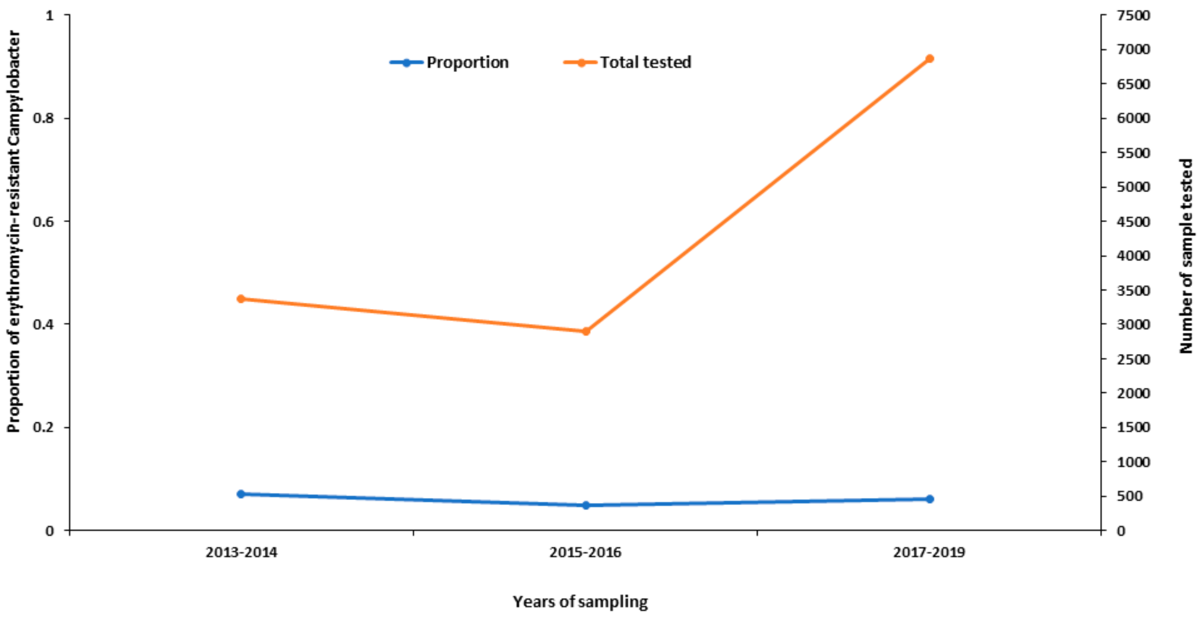

Fig. S5. Temporal trends in the proportion of erythromycin-resistant *Campylobacter* spp. by years of sampling

Table S1. Univariable logistic regression of association between years of sampling and tetracycline-resistant *Salmonella* spp. isolates in cecal samples of food-producing animals in the United States

Variable	Categories	OR	95% CI	p-value
**Years of sampling** (n= 8,968)				0.0183
	2013-2014 vs. 2017-2019	0.85	0.74, 0.97	0.0132
	2015-2016 vs. 2017-2019	0.94	0.83, 1.08	0.5620
	2013-2014 vs. 2015-2016	0.90	0.77, 1.06	0.2615

OR — Odds ratio; CI — Confidence interval

Table S2. Univariable logistic regression of association between years of sampling and tetracycline-resistant *Campylobacter* spp. isolates in cecal samples of food-producing animals in the United States

Variable	Categories	OR	95% CI	p-value
**Years of sampling** (n= 13,160)				<0.0001
	2013-2014 vs. 2017-2019	1.27	1.14, 1.41	<0.0001
	2015-2016 vs. 2017-2019	1.26	1.13, 1.41	<0.0001
	2013-2014 vs. 2015-2016	1.00	0.88, 1.14	0.9979

OR — Odds ratio; CI — Confidence interval

Table S3. Univariable logistic regression of association between years of sampling and tetracycline-resistant *Escherichia coli* isolates in cecal samples of food-producing animals in the United States

Variable	Categories	OR	95% CI	p-value
**Years of sampling** (n= 12,618)				0.1584
	2013-2014 vs. 2017-2019	0.96	0.85, 1.09	0.7645
	2015-2016 vs. 2017-2019	1.07	0.97, 1.17	0.2694
	2013-2014 vs. 2015-2016	0.904	0.79, 1.04	0.1972

OR — Odds ratio; CI — Confidence interval

Table S4. Univariable logistic regression of association between years of sampling and erythromycin-resistant *Campylobacter* spp. isolates in cecal samples of food-producing animals in the United States

Variable	Categories	OR	95% CI	p-value
**Years of sampling** (n= 13,160)				0.0026
	2013-2014 vs. 2017-2019	1.18	0.97, 1.44	0.1205
	2015-2016 vs. 2017-2019	0.81	0.64, 1.03	0.0894
	2013-2014 vs. 2015-2016	1.46	1.13, 1.89	0.0017

OR — Odds ratio; CI — Confidence interval
